# DNA metabarcoding of fungal diversity in air and snow of Livingston Island, South Shetland Islands, Antarctica

**DOI:** 10.1038/s41598-020-78630-6

**Published:** 2020-12-11

**Authors:** Luiz Henrique Rosa, Otávio Henrique Bezerra Pinto, Tina Šantl-Temkiv, Peter Convey, Micheline Carvalho-Silva, Carlos Augusto Rosa, Paulo E. A. S. Câmara

**Affiliations:** 1 Laboratório de Microbiologia Polar e Conexões Tropicais, Departamento de Microbiologia, Instituto de Ciências Biológicas, Universidade Federal de Minas Gerais, P.O. Box 486, Belo Horizonte, MG CEP 31270-901 Brazil; 2 Departamento de Biologia Celular, Universidade de Brasília, Brasília, Brazil; 3 Department of Bioscience, Aarhus University, Building 1540 Office 124, 116 Ny Munkegade, 8000 Aarhus C, Denmark; 4 British Antarctic Survey, NERC, High Cross, Madingley Road, Cambridge, CB3 0ET UK; 5 Departamento de Botânica, Universidade de Brasília, Brasília, Brazil

**Keywords:** Environmental microbiology, Fungi, Fungal ecology

## Abstract

We assessed fungal diversity present in air and freshly deposited snow samples obtained from Livingston Island, Antarctica, using DNA metabarcoding through high throughput sequencing (HTS). A total of 740 m^3^ of air were pumped through a 0.22 µm membrane. Snow obtained shortly after deposition was kept at room temperature and yielded 3.760 L of water, which was filtered using Sterivex membranes of 0.22 µm mesh size. The total DNA present was extracted and sequenced. We detected 171 fungal amplicon sequence variants (ASVs), 70 from the air and 142 from the snow. They were dominated by the phyla *Ascomycota*, *Basidiomycota*, *Mortierellomycota* and *Mucoromycota*. *Pseudogymnoascus*, *Cladosporium*, *Mortierella* and *Penicillium* sp. were the most dominant ASVs detected in the air in rank order. In snow, *Cladosporium*, *Pseudogymnoascus*, *Penicillium*, *Meyerozyma*, *Lecidea*, *Malassezia*, *Hanseniaspora*, *Austroplaca*, *Mortierella*, *Rhodotorula*, *Penicillium*, *Thelebolus*, *Aspergillus*, *Poaceicola*, *Glarea* and *Lecanora* were the dominant ASVs present. In general, the two fungal assemblages displayed high diversity, richness, and dominance indices, with the assemblage found in snow having the highest diversity indices. Of the total fungal ASVs detected, 29 were only present in the air sample and 101 in the snow sample, with only 41 present in both samples; however, when only the dominant taxa from both samples were compared none occurred only in the air and, among the rare portion, 26 taxa occurred in both air and snow. Application of HTS revealed the presence of a more diverse fungal community in the air and snow of Livingston Island in comparison with studies using traditional isolation methods. The assemblages were dominated by cold-adapted and cosmopolitan fungal taxa, including members of the genera *Pseudogymnoascus*, *Malassezia* and *Rhodotorula*, which include some taxa reported as opportunistic. Our results support the hypothesis that the presence of microbiota in the airspora indicates the possibility of dispersal around Antarctica in the air column. However, further aeromycology studies are required to understand the dynamics of fungal dispersal within and beyond Antarctica.

## Introduction

Antarctica represents one of the most pristine regions of the planet and, despite the multiple extreme conditions that characterize it, harbours a considerable terrestrial biodiversity, mainly of microorganisms, that are able to survive and colonize its different environments. Due the continent’s isolation from lower latitudes by the oceanic Antarctic Circumpolar Current and atmospheric circulation, the lack of trophic complexity, and the vulnerability of its endemic biodiversity to environmental changes and anthropogenic influences, Antarctica provides a unique opportunity for microbial aerobiology studies seeking to understand how airspora are transported to and within Antarctica^1,2^. The extent to which Antarctic environments receive microbial propagules, potentially including globally cosmopolitan species from outside Antarctica, remains largely unstudied, although they have been detected in the air column and after deposition, for instance in snow and ice^3–7^. According to Archer et al*.*^2^, microbial communities present in ecosystems of isolated regions of Antarctica, such as the Victoria Land Dry Valleys, display limited connectivity to the global microbial pool due the strong selection that occurs during atmospheric transport, resulting in regionally isolated airborne inputs and highly specialized soil communities, with fungi also displaying greater isolation from non-polar sources than bacteria. However, detailed information about the aerial routes by which microorganisms arrive and circulate in Antarctica is lacking^8,9^.

Biological dispersal by aerial means can be an important factor shaping patterns of biodiversity^9,10^. Viable organisms or their propagules present in the air column may be in dormant and cryptobiotic states, where they are metabolically inactive due the harsh dry, cold, low nutrient and high irradiance conditions. Diverse groups of microorganisms have been recorded in the few Antarctic aerobiological studies completed to date (reviewed by Pearce et al*.*^9^), including viruses, bacteria, microalgae and fungi.

Mycological studies in Antarctica have shown that much of the Antarctic fungal community is represented by cold tolerant (psychrophilic or psychrotolerant) species, many of which have wide and even globally cosmopolitan distributions, with presence in polar, temperate, and tropical environments^11^. de Menezes et al*.*^12^ suggested that the high densities of cosmopolitan fungi present in snow are consistent with them being present in air masses arriving at the Antarctic Peninsula from beyond Antarctica, which are then entrained in snow precipitation, and become concentrated in the snow. Snow and ice can provide an indirect record of the presence and deposition of fungal propagules (e.g. spores or hyphal fragments) from the air column over time^12^. In snow samples obtained from six different regions of the Antarctic Peninsula, de Menezes et al*.*^13^ reported a rich fungal diversity assigned to 51 species in 26 genera and dominated by cold tolerant cosmopolitan fungi. However, in ice from continental Antarctica and the Antarctic Peninsula, Rogers et al*.*^14^ and de Menezes et al*.*^15^, respectively, reported much lower fungal diversity. In the present study, we assessed fungal diversity present in air and freshly deposited snow samples obtained from Livingston Island, Antarctica, using DNA metabarcoding through high-throughput sequencing (HTS).

## Material and methods

### Snow and air sampling

**Figure 1:**
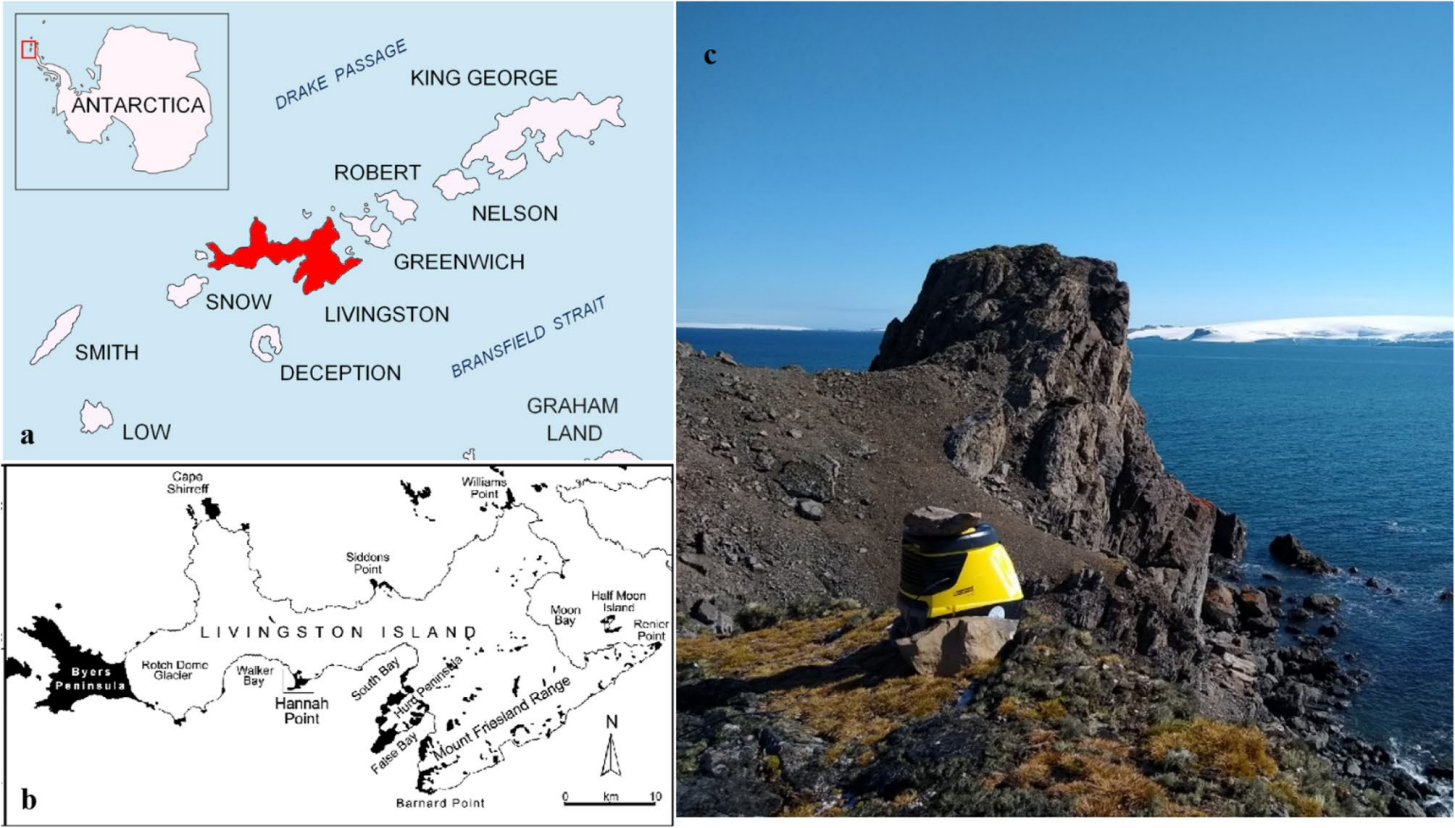
Location of soil sample collections. (**a**) Antarctic Peninsula, (**b**) Livingston Island and (**c**) Punta Polaca at Hurd Peninsula, where the air and snow were sampled [62°40′16″ S; 60°22′43″ W]. Photo (**c**) by T Šantl-Temkiv.

Air and snow samples were collected at Punta Polaca (62°40′16″ S; 60°22′43″ W), Hurd Peninsula, Livingston Island, South Shetland Islands, near to the Spanish station Juan Carlos I (Fig. 1). Two air samples were collected with a high flow glass impinger following Šantl-Temkiv et al*.*^16,17^. The chamber was filled with 2 L of sampling liquid (ddH_2_O) and the sampler was run for 5 min, so that the liquid came in contact with the entire chamber, after which 0.5 L of the sampling liquid was removed, stored as a control, and analyzed along with the samples. The control represented a field blank to certify that the samples were not contaminated by external organisms. The resulting solution was filtered directly on the Sterivex filter units for the air, as described by Lever et al*.*^18^. Air was collected over c. 5 h on March 11th 2019. In addition, the two separate air DNA extractions were combined together in order to increase DNA yield. Two freshly deposited snow samples were collected on March 20^th^ 2019 at the same site using a sterilized shovel. Both pairs of samples were separately combined in order to increase DNA yield. Snow was melted at room temperature, under strictly sterile conditions, for 24 h in the laboratory at Juan Carlos I Station and then filtered using Sterivex filters^18^.

### DNA extraction and data analysis

Total DNA was extracted from environmental samples using the Qiagen Power Soil Kit (Qiagen, USA) following the manufacturer’s instructions. Extracted DNA was used as template for generating PCR amplicons. The internal transcribed spacer 2 (ITS2) of the nuclear ribosomal DNA was used as a DNA barcode for molecular species identification^19,20^. PCR amplicons were generated using the universal primers ITS3 and ITS4^21^ and were sequenced by high-throughput sequencing at Macrogen Inc. (South Korea) on an Illumina MiSeq sequencer, using the MiSeq Reagent Kit v3 (600-cycle) following the manufacturer’s protocol.

Raw fastq files were filtered using BBDuk version 38.34 (BBMap—Bushnell B.—sourceforge.net/projects/bbmap/) to remove Illumina adapters, known Illumina artifacts, and PhiX Control v3 Library. Quality read filtering was carried out using Sickle version 1.33-q 30-l 50^22^, to trim ends 3′ or 5′ with low Phred quality score. Sequences shorter than 50 bases were discarded. These sequences were imported to QIIME2 version 2019.10 (https://qiime2.org/) for bioinformatics analyses^23^. The qiime2-dada2 plugin is a complete pipeline that was used for filtering, dereplication, turning paired-end fastq files into merged reads, and removal of chimeras^24^. Taxonomic assignment was carried out for the amplicon sequence variants (ASVs) using qiime2-feature-classifier^25^ classify-sklearn against the UNITE fungal ITS database version 7.2^26^ and trained with Naive Bayes classifier. A confidence threshold of 98.5% was used. All raw sequences have been deposited in the NCBI database under the codes SRR12830238, SRR12830240 and SRR12830239.

Many factors, including extraction, PCR, and primer bias, can affect the number of reads^27^, and thus lead to misinterpretation of abundance^28^. However, Giner et al*.*^29^ concluded that such biases did not affect the proportionality between reads and cell abundance, implying that more reads are linked with higher abundance^29,30^. Therefore, for comparative purposes we used the number of reads as a proxy for relative abundance.

All sequences obtained from air and snow samples were matched with sequences present in the list of the top 50 ‘most wanted’ fungi according to Nilsson et al*.*^31^. The sequences were merged, filtered, dereplicated, and clustered into > 97% identity ASVs using USEARCH version 10^32^. Nucleotide-Nucleotide BLAST 2.6.0 + was used to compare these ASVs against the top50_release_04.02.2020.fasta^33^, considering just subject matches with aligned length longer than 250 bp and > 98% identity.

### Fungal diversity and distribution

To quantify species diversity, richness, and dominance, we used the following indices: (i) Fisher’s α, (ii) Margalef’s, and (iii) Simpson’s, respectively. The numbers of DNA reads of the amplicon sequence variants (ASVs) were used to quantify the fungal taxa present in the air sampled, where fungal ASVs with more than 1,000 reads were considered dominant and < 1,000 minor components (rare) of the fungal community. All of the results were obtained with 95% confidence, and bootstrap values were calculated from 1,000 iterations. Taxon species accumulation curves were obtained using the Mao Tao index. All diversity indices and species accumulation curves calculations were performed using PAST v. 1.90^34^. Venn diagrams were prepared according to Bardou et al*.*^35^ to compare the fungal assemblages present in both air and snow samples. The functional assignments of fungal ASVs at species and genera levels are shown using FunGuild^36^.

## Results

### Fungal taxonomy

**Figure 2:**
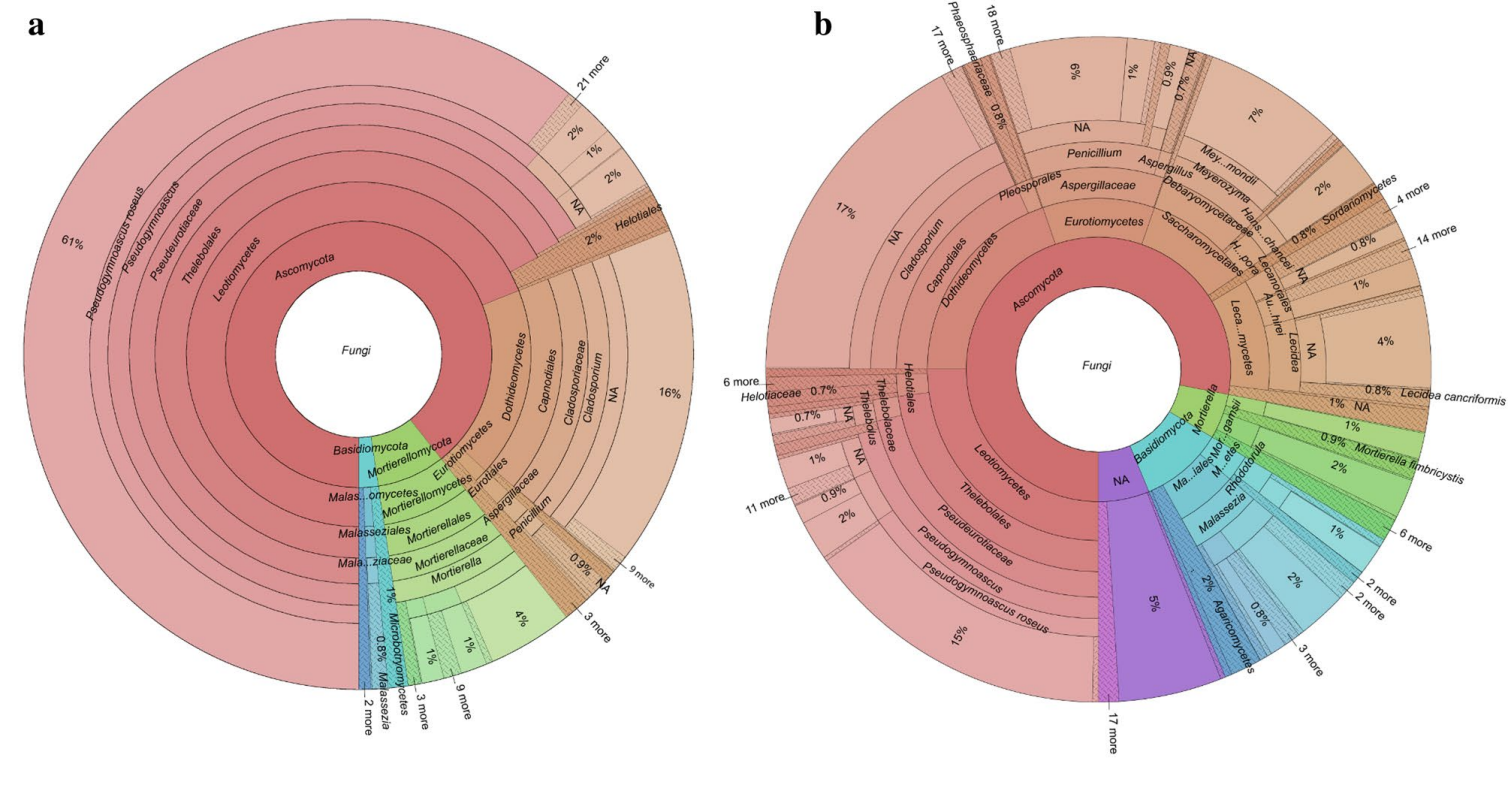
Krona chart of (**a**) fungal assemblages detected in the air and (**b**) in snow from Livingston Island, South Shetland Islands, Antarctica.

The number of reads in the air sample was 162,038 and that in snow 268,710. From these, we detected 171 fungal amplicon sequence variants (ASVs), 70 in 740 m^3^ of air and 142 in 3.76 L of snow from Livingston Island, Antarctica (Table 1; Fig. 2). The ASVs were dominated by the phyla *Ascomycota*, *Basidiomycota* and *Mortierellomycota*. In the air sample, ASVs identified as *Pseudogymnoascus roseus*, *Cladosporium* sp., *Mortierella* sp. 1, *Pseudogymnoascus* sp. 3, *Pseudogymnoascus* sp. 2, *Mortierella fimbricystis*, *Mortierella gamsii* and *Penicillium* sp. were the most dominant taxa (all with > 1,000 reads), in rank order. In contrast, 27 fungal ASVs (*Cladosporium* sp., *Pseudogymnoascus roseus*, *Penicillium* sp., *Meyerozyma guilliermondii*, *Lecidea* sp., *Malassezia restricta*, *Pseudogymnoascus* sp. 3, *Hanseniaspora lachancei*, *Pseudogymnoascus* sp. 2, *Austroplaca darbishirei*, *Mortierella gamsii*, *Malassezia globosa*, *Rhodotorula diobovata*, *Mortierella* sp. 1, *Ascomycota* sp., *Mortierella fimbricystis*, *Penicillium polonicum*, *Lecanorales* sp., *Thelebolus* sp., *Lecidea cancriformis*, *Aspergillus* sp., *Poaceicola agrostina*, *Glarea* sp., *Pseudogymnoascus* sp. 1, *Mortierella* sp. 2, *Thelebolus globosus* and *Lecanora physciella*) were present as dominant fungi in snow. A further 177 ASVs (62 in air and 115 in snow) were detected less frequently (< 1,000 reads) and may represent the rare portion of the fungal assemblages. In addition, 78 ASVs could only be assigned to higher taxonomic levels (phylum, class, order or family). A total of 29,069 sequences from the air and 6,223 from the snow samples were matched with the sequences of 11 unidentified species hypotheses in the list of the top 50 most wanted fungi^31^ with the alignment length longer than 250 bp and > 98% identity (Suppl. Table 1).

**Table 1:** Numbers of sequence reads of fungal amplicon sequence variants (ASVs) detected in air and snow samples from Livingston Island, South Shetlands, Antarctica.

Hierarchical level	Fungal taxa (ASVs)*	Reference sequences	Reads by Samples		Total
			Air	Snow	
Fungi	Fungi sp.		39**	20,958	20,997
*Ascomycota*	*Pseudogymnoascus roseus*	SH1557165.08FU	61,935	0	61,935
	*Cladosporium* sp.	SH1521536.08FU	20,801	0	20,801
	*Pseudogymnoascus* sp.	SH1557215.08FU	2,035	1,5650	17,685
	*Meyerozyma* sp.	SH1516625.08FU	0	1,5735	15,735
	*Penicillium* sp.	SH1530043.08FU	431	9,385	9,816
	*Lecidea cancriformis*	SH2711223.08FU	0	6,781	6,781
	*Hanseniaspora* sp.	SH1547214.08FU	0	4,708	4,708
	*Austroplaca darbishirei*	SH1633428.08FU	0	3,165	3,165
	*Thelebolus globosus*	SH1647628.08FU	271	1,614	1,885
	*Helotiales* sp.	SH1648813.08FU	1,075	404	1,479
	*Penicillium polonicum*	SH1529888.08FU	0	1,233	1,233
	*Pseudogymnoascus appendiculatus*	SH1939321.08FU	1138	0	1,138
	*Septoriella* sp.	SH1525156.08FU	0	902	902
	*Lecanora physciella*	SH1636780.08FU	0	738	738
	*Cyberlindnera* sp.	SH1648567.08FU	571	0	571
	*Mitrulinia* sp.	SH1574181.08FU	0	482	482
	*Cleistothelebolus nipigonensis*	SH1630064.08FU	0	433	433
	*Chalara pseudoaffinis*	SH1522386.08FU	368	0	368
	*Pestalotiopsis* sp.	SH1562655.08FU	0	364	364
	*Neoascochyta paspali*	SH1547057.08FU	329	4	333
	*Paraconiothyrium africanum*	SH1525457.08FU	0	331	331
	*Debaryomyces* sp.	SH1516581.08FU	62	251	313
	*Phaeoacremonium hungaricum*	SH1644597.08FU	0	287	287
	*Lecidea* sp.	SH1524770.08FU	0	277	277
	*Colletotrichum* sp.	SH1636843.08FU	186	90	276
	*Rhizoscyphus* sp.	SH1543082.08FU	169	103	272
	*Aspergillus* sp.	SH1536361.08FU	0	249	249
	*Schwanniomyces polymorphus*	SH1649127.08FU	0	244	244
	*Septoriella hirta*	SH2714710.08FU	0	225	225
	*Ascomycota* sp.	SH1574206.08FU	123	82	205
	*Penicillium fluviserpens*	SH1536160.08FU	0	199	199
	*Saccharomyces cerevisiae*	SH1583301.08FU	0	193	193
	*Aspergillus niger*	SH3322875.08FU	0	183	183
	*Volucrispora graminea*	SH1605412.08FU	0	154	154
	*Aspergillus sydowii*	SH1550060.08FU	38	113	151
	*Penicillium steckii*	SH1692788.08FU	0	150	150
	*Leptosphaeria sclerotioides*	SH1624038.08FU	147	0	147
	*Leotiomycetes* sp.	SH1647738.08FU	136	0	136
	*Pseudallescheria* sp.	SH2328594.08FU	0	132	132
	*Buellia russa*	SH1551132.08FU	0	130	130
	*Chaetothyriales* sp.	SH1545109.08FU	0	129	129
	*Penicillium brasilianum*	SH1692798.08FU	0	123	123
	*Phaeosphaeria dennisiana*	SH1530704.08FU	120	0	120
	*Pseudallescheria ellipsoidea*	SH2328455.08FU	0	112	112
	*Lodderomyces elongisporus*	SH1507873.08FU	103	0	103
	*Candida tropicalis*	SH1542296.08FU	101	0	101
	*Yamadazyma* sp.	SH1539910.08FU	101	0	101
	*Trichoderma* sp.	SH1542292.08FU	0	91	91
	*Didymellaceae* sp.	SH1547074.08FU	82	0	82
	*Penicillium paxilli*	SH1530009.08FU	8	73	81
	*Parmeliaceae* sp.	SH1541255.08FU	71	0	71
	*Paraphoma fimeti*	SH1616190.08FU	0	70	70
	*Colletotrichum annellatum*	SH2219599.08FU	0	67	67
	*Polysporina subfuscescens*	SH1596449.08FU	0	67	67
	*Pseudeurotium* sp.	SH3332798.08FU	67	0	67
	*Dermateaceae* sp.	SH1522957.08FU	66	0	66
	*Penicillium astrolabium*	SH1530010.08FU	0	66	66
	*Cladosporium halotolerans*	SH1525346.08FU	37	27	64
	*Diaporthales* sp.	SH1657193.08FU	64	0	64
	*Lecanoromycetes* sp.	SH1517968.08FU	0	60	60
	*Lecanora contractula*	SH1527996.08FU	0	55	55
	*Ramalinaceae* sp.	SH1522446.08FU	0	51	51
	*Cystodendron* sp.	SH1524864.08FU	50	0	50
	*Penicillium cairnsense*	SH2190109.08FU	0	50	50
	*Cladonia rei*	SH3326345.08FU	49	0	49
	*Neodevriesia capensis*	SH3331962.08FU	0	49	49
	*Neopestalotiopsis* sp.	SH3324784.08FU	49	0	49
	*Penicillium sumatraense*	SH1585145.08FU	9	37	46
	*Mycosphaerella tassiana*	SH1607937.08FU	0	44	44
	*Pseudeurotiaceae* sp.	SH1556184.08FU	44	0	44
	*Fusarium solani*	SH2721166.08FU	43	0	43
	*Placopsis contortuplicata*	SH1521544.08FU	0	40	40
	*Schwanniomyces* sp.	SH2154634.08FU	38	0	38
	*Bacidina arnoldiana*	SH3321741.08FU	0	28	28
	*Penicillium citrinum*	SH1539276.08FU	15	13	28
	*Zymoseptoria verkleyi*	SH1544001.08FU	21	0	21
	*Sarocladium* sp.	SH1542060.08FU	17	0	17
	*Aspergillus penicillioides*	SH1537266.08FU	16	0	16
	*Pichia kluyveri*	SH1527730.08FU	16	0	16
	*Botryosphaeriaceae* sp.	SH3317647.08FU	0	6	6
	*Fusarium asiaticum*	SH2456121.08FU	0	4	4
	*Usnea* sp.	SH1550545.08FU	0	3	3
*Basidiomycota*	*Malassezia restricta*	SH2734004.08FU	401	4,740	5,141
	*Malassezia globosa*	SH1565779.08FU	165	2,946	3,111
	*Rhodotorula diobovata*	SH1585138.08FU	0	3,060	3,060
	*Agaricomycetes* sp.	SH1575746.08FU	0	2,581	2,581
	*Malassezia* sp.	SH1546915.08FU	22	1,548	1,570
	*Marasmius* sp.	SH1514868.08FU	912	0	912
	*Rhodotorula mucilaginosa*	SH1558606.08FU	750	120	870
	*Leucosporidiella creatinivora*	SH1651377.08FU	404	0	404
	*Heterochaete shearii*	SH1561152.08FU	75	259	334
	*Malasseziales* sp.	SH1547455.08FU	46	266	312
	*Calyptella capula*	SH1635872.08FU	0	170	170
	*Pluteus ephebeus*	SH2724840.08FU	158	0	158
	*Malassezia equina*	SH2723257.08FU	0	95	95
	*Vishniacozyma victoriae*	SH1572254.08FU	94	0	94
	*Phanerochaete sordida*	SH1573517.08FU	83	0	83
	*Hyphodontia microspora*	SH1651385.08FU	82	0	82
	*Peniophora laxitexta*	SH1646415.08FU	56	0	56
	*Gymnopus* sp.	SH1560298.08FU	50	0	50
	*Vishniacozyma tephrensis*	SH1691243.08FU	48	0	48
	*Microbotryomycetes* sp.	SH2750674.08FU	40	0	40
	*Vanrija humicola*	SH1514178.08FU	30	0	30
	*Basidiomycota* sp.	SH1514435.08FU	0	19	19
	*Polyporales* sp.	SH1651381.08FU	15	0	15
	*Malassezia sympodialis*	SH3313592.08FU	0	12	12
*Mortierellomycota*	*Mortierella* sp.	SH1557435.08FU	5,878	744	6,622
	*Mortierella fimbricystis*	SH2452854.08FU	2,260	0	2,260
	*Mortierella gamsii*	SH1556972.08FU	1,416	155	1,571
	*Mortierella parvispora*	SH1629873.08FU	396	0	396
	*Mortierella alpina*	SH1503809.08FU	158	0	158
	*Mortierella elongatula*	SH1574597.08FU	0	74	74
	*Mortierella turficola*	SH3338068.08FU	0	56	56
*Mucoromycota*	*Densospora* sp.	SH3319965.08FU	0	145	145

*ASVs = amplicon sequence variants; **number of the reads.

### Fungal diversity

**Figure 3:**
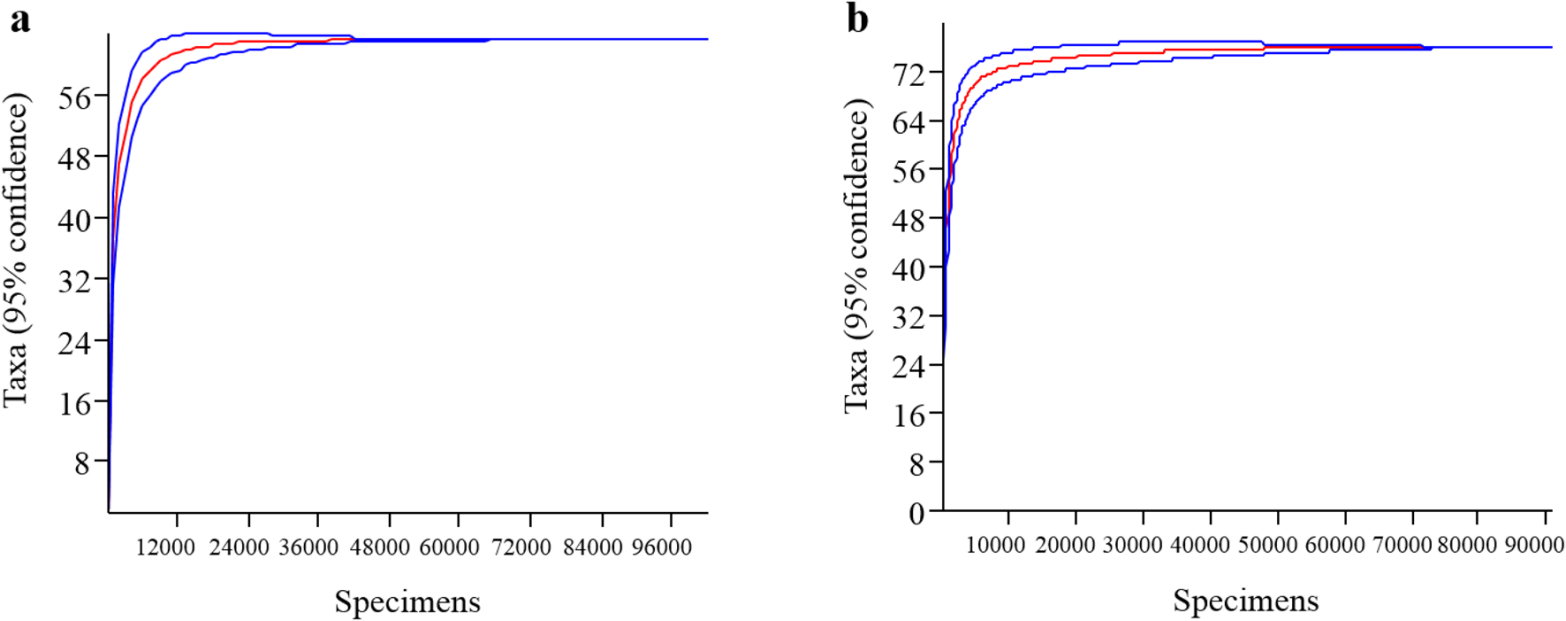
Rarefaction curves for samples from fungal assemblages present in the **(a)** air and **(b)** snow on Livingston Island, South Shetlands, Antarctica. Blue lines represent confidence limits inferred using bootstrap values calculated from 1,000 iterations using PAST, version 1.90^34^.

**Figure 4:**
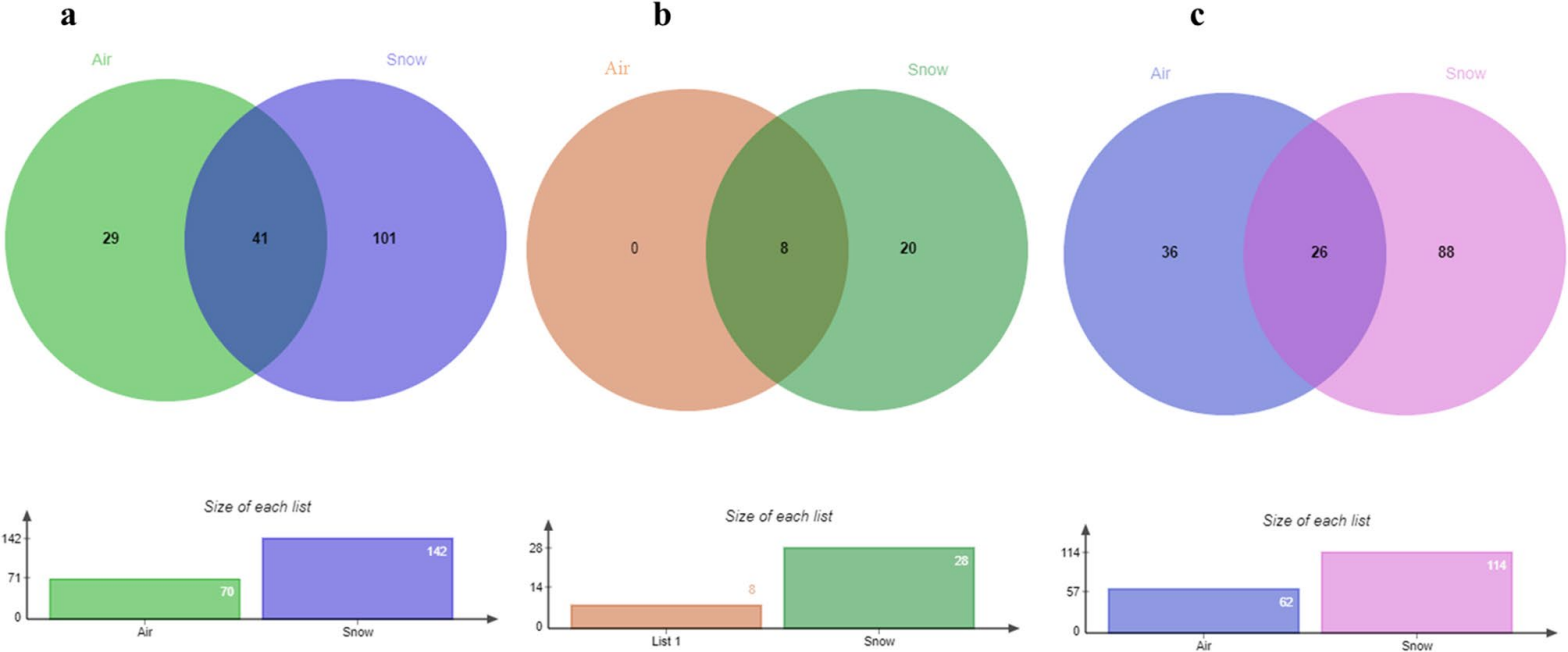
(**a**) Venn diagram showing the (**a**) total, (**b**) dominant (those with > 1,000 reads) and (**c**) rare fungal taxa distribution detected in air and snow of Livingston Island, South Shetlands, Antarctica.

The Mao Tao rarefaction curves of the fungal assemblages present in air and snow reached asymptote for both fungal assemblages (Fig. 3), indicating that the data provided a good description of the diversity present. In general, both fungal assemblages displayed high diversity, richness, and dominance indices (Table 2). The assemblage present in the snow was more diverse, rich, and included a wider range of dominant fungi when compared with that from the air sample. Of the total fungal ASVs detected, 29 were only present in the air sample and 101 in the snow sample, with 41 present in both samples (Fig. 4a). However, when only the dominant ASVs (> 1,000 reads) from both samples were compared, none occurred only in the air (Fig. 4b) and, among the rare portion, 26 occurred in both air and snow (Fig. 4c). In addition, the ecological functional assignments of fungal ASVs in species and genera levels were showed in Suppl. Table 2 and Suppl. Table 3, respectively.

**Table 2:** Sample data and ecological indices of the fungal DNA recovered from air and snow samples from Livingston Island, South Shetlands, Antarctica.

Ecological indices	Sample		
	Air	Snow	Total
Number of reads	162,038	268,710	430,748
Number of taxa	70	142	171
Fisher α	6.96	14.44	16.85
Margalef	5.75	11.3	13
Simpson	0.6	0.92	0.85

## Discussion

### Fungal taxonomy and diversity

Despite an increase in mycological studies, fungal diversity in Antarctica remains poorly known^11^. According to Bridge and Spooner^37^, around 1,000 fungal species have been described from Antarctica, identified using a range of approaches including traditional methods for cultivable fungi such as macro- and/or micromorphology of colonies and fruiting bodies as well as DNA sequencing of mycelia of cultivable fungi. Most airborne mycological studies in Antarctica have relied on traditional morphological methods. Marshall^4^ monitored airborne fungal spores over 13.6 months at three sites on Signy Island (South Orkney Islands) in the maritime Antarctic, reporting that *Epicoccum* spp. and *Cladosporium* spp. dominated the diversity present. Duncan et al*.*^38^ sampled air inside the historic wooden huts on Ross Island, finding *Cladosporium cladosporioides*, *Pseudeurotium desertorum*, *Pseudogymnoascus* sp. and *Antarctomyces psychrotrophicus* as dominant viable fungal propagules and *Cadophora* sp. and *Thebolus* sp. as minor components of the outdoor airborne fungal assemblage. Archer et al*.*^2^ compared microbial diversity in near-ground and high-altitude air above the Victoria Land Dry Valleys as well as that of underlying soil microbial communities, finding basidiomycete yeasts to be dominant in the air and unclassified fungi to be common in soils. However, the more recent fungal inventories using metabarcoding approaches have demonstrated that fungal diversity in Antarctica is greater than previously recognised^39–41^.

As air and snow are typically ultra-oligotrophic microhabitats, few viable fungal taxa are expected to be present, as reported by de Menezes et al*.*^12^ who, using cultivation techniques, reported only 14 fungal taxa in snow samples from several different Antarctic islands. However, despite analysing only a small a small absolute sample size of air and snow collected in the Livingston Island, use of the HTS approach in the current study revealed the presence of much greater fungal diversity in both air and snow, many of which display mechanisms that render them well-adapted to survive atmospheric transport, such as the production of resistant spores and UV protective compounds^42,43^.

The dominant taxa detected in the air included representatives of *Pseudogymnoascus*, *Cladosporium*, *Mortierella* and *Penicillium*. However, even though recently deposited snow would be expected to contain microbial airborne particles entrained from the air column as the snow fell, fungal diversity in the snow sampled was very different to that in the air over the same location. In snow sample, the dominant taxa found included representatives of *Cladosporium*, *Pseudogymnoascus*, *Penicillium*, *Meyerozyma*, *Lecidea*, *Malassezia*, *Hanseniaspora*, *Austroplaca*, *Mortierella*, *Rhodotorula*, *Penicillium*, *Thelebolus*, *Aspergillus*, *Poaceicola*, *Glarea* and *Lecanora*. The diversity present in both the air and snow samples also included dominant taxa that could only be assigned to higher taxonomic levels such as Fungal sp., *Ascomycota* sp., *Basidiomycota* sp., *Agaricales* sp., *Chaetothyriales* sp., *Helotiales* sp., *Lecanorales* sp. and *Polyporales* sp. These may represent currently undescribed or otherwise unsequenced species, further supporting the assertion that much of the true fungal diversity present in Antarctica is currently unknown.

*Pseudogymnoascus* were detected as dominant fungi in both air and snow samples. *Pseudogymnoascus* (previously known as *Geomyces*) is a genus often detected in cold environments including those of polar, alpine, and temperate regions^11,44–47^. In Antarctica, it has been reported from soils^44,48–50^, associated with plants^51–54^ and macroalgae^55^, in freshwater lakes^56^, and associated with lichens^57^. *Cladosporium* and *Penicillium* also represent common airborne fungi reported globally, including Antarctica. *Cladosporium* is a dematiaceous fungal group with global distribution^58^. In Antarctic microhabitats, *Cladosporium* has mainly been detected in association with plants and soil^11^. *Penicillium* is a ubiquitous genus, again detected in multiple substrates in Antarctica including soils^50,59,60^, permafrost^61,62^ and associated with macroalgae^63^. The abundant presence of *Pseudogymnoascus*, *Cladosporium*, and *Penicillium* both in air and snow sampled indicated that these fungi may circulate at least around the Antarctic Peninsula.

The genus *Mortierella* includes about 85 species, which occur mainly in soils^64^. *Mortierella* species are found worldwide, particularly in temperate and polar regions. Representatives of the genus are abundant in Antarctica and reported in association with plants^51,52^, macroalgae^63^, lichens^57^, soils^65^, freshwater^56^, and permafrost^62^. Some species of *Mortierella* are known as snow moulds and have the capability to growth and produce spores at 0°C^66^. They occur abundantly in the interstitial water in Antarctic snow where snow melting occurs in summer, for instance in association with snow algal communities.

The genus *Malassezia* includes 17 species of basidiomycetous pigmented black yeast species generally present in the skin and mucosa microbiome of humans and other warm-blooded animals^67^. According to Prohic et al*.*^68^, several *Malassezia* species found on human and animal skin are commensals, but they can also be associated with *Pityriasis versicolor*, *Malassezia folliculitis*, seborrheic dermatitis/dandruff, atopic dermatitis, and psoriasis. The detection of *Malassezia* in Antarctica is unusual. Rosa et al*.*^54^ detected different *Malassezia* taxa in soil samples from undisturbed and disturbed (by human activity) sites on Deception Island (South Shetland Islands) using HTS metabarcoding techniques.

The genus *Meyerozyma* includes species that are typically widely distributed or cosmopolitan^69^. Species of *Meyerozyma* have previously been isolated from aquatic environments in Antarctica^69,70^ and associated with macroalgae^63^. The genus *Hanseniaspora* (anamorph *Kloeckera*) includes ascomycete yeast species commonly associated with alcoholic fermentation, but is also recorded from soil, plants, fruit-eating insects, birds, and seafood^71^. Some *Hanseniaspora* species have been reported as unusual opportunistic superficial mycosis in humans^72–75^.

The genus *Rhodotorula* includes cosmopolitan pigmented yeast species and is often dominant in extreme environments^76^, including those of Antarctica^63,70^. Our study represents the first report of high abundance of *R. muscilagionsa* in Antarctic snow samples, although de Menezes et al*.*^13^ reported the species among the dominant fungi detected in snow samples from several Antarctic islands. The genus *Thelebolus* is distributed globally and representatives occur in diverse habitats^77^. Species of *Thelebolus* have been reported in Arctic and Antarctic environments^78,79^, as being abundant in lakes, and in association with birds (skuas)^80^, in freshwater^56,81^ and in ice^15^. Finally, from the air and snow sampled in Livingston Island, Antarctica, we detected 11 unidentified species hypotheses in the list of the top 50 most wanted fungi^31^, suggesting the both habitats may shelter rare species that merit further taxonomic attention.

## Conclusions

We used DNA metabarcoding to catalogue the fungi present in air and snow samples from Livingston Island, South Shetland Islands. This revealed a diverse fungal community comprising taxa from the phyla *Ascomycota*, *Basidiomycota*, *Mortierellomycota* and *Mucoromycota*. The assemblages were dominated by cold-adapted and cosmopolitan (psychrophilic) taxa, including members of the genera *Pseudogymnoascus*, *Malassezia* and *Rhodotorula*, which include taxa reported as opportunistic fungi. Our results confirm the presence of fungi in the airspora, supporting the possibility of dispersal over different geographical scales around Antarctica in the air column. Given that many of the taxa identified in this study are known from Antarctic fungal communities, a local source for those present in the air column is plausible. The large proportion of unassigned taxa highlight the poor level of baseline knowledge of Antarctic fungal diversity, and further aeromicrobiology and diversity studies are required to understand the dynamics of fungal dispersal within and beyond Antarctica. However, as metabarcoding detects environmental DNA, the technique can also detect DNA from dead fungi or otherwise non-viable material. Further studies will be necessary to develop strategies to isolate these fungi into culture.

## Supplementary information

Supplementary Information.

Supplementary Information.

Supplementary Information.

Supplementary References.
