# Supplementary material for: DNA metabarcoding of fungal diversity in air and snow of Livingston Island, South Shetland Islands, Antarctica
Source: Sci Rep. 2020 Dec 11;10:21793. doi: 10.1038/s41598-020-78630-6 (PMC7733504; doi:10.1038/s41598-020-78630-6)
Supplement: Supplementary file 1 — Supplementary Information. [file 41598_2020_78630_MOESM1_ESM.docx]

**DNA metabarcoding of fungal diversity in air and snow of Livingston Island, South Shetland Islands, Antarctica**

Luiz Henrique Rosa, Otávio Henrique Bezerra Pinto, Tina Santl-Temkiv, Peter Convey, Micheline Carvalho-Silva, Carlos Augusto Rosa and Paulo EAS Câmara

**Supplementary Table 1.** Number of amplicon sequence variants assigned to the top 50 most wanted fungi.

|  | Number of ASVs* | |
| --- | --- | --- |
| Reference sequences | Air | Snow |
| SH1240070.08FU | 330 | 1,797 |
| SH1241071.08FU | 28,306 | 39 |
| SH1243585.08FU | 63 | 0 |
| SH1239438.08FU | 370 | 1,319 |
| SH1143655.08FU | 0 | 421 |
| SH1239292.08FU | 0 | 76 |
| SH1242249.08FU | 0 | 255 |
| SH1187739.08FU | 0 | 133 |
| SH1157237.08FU | 0 | 2,166 |
| SH1143093.08FU | 0 | 11 |
| SH1143317.08FU | 0 | 6 |
| **Total** | **29,069** | **6,223** |

*ASVs = amplicon sequence variants. The sequences were BLASTN-searched against the latest version of the Fasta file containing the top 50 most wanted fungi, i.e., top50_release_04.02.2020.fasta. The numbers of the sequences aligned with the subject sequences with the aligned length longer than 250 bases and 98% identity are shown.
