# Supplementary material for: DNA metabarcoding of fungal diversity in air and snow of Livingston Island, South Shetland Islands, Antarctica
Source: Sci Rep. 2020 Dec 11;10:21793. doi: 10.1038/s41598-020-78630-6 (PMC7733504; doi:10.1038/s41598-020-78630-6)
Supplement: Supplementary file 2 — Supplementary Information. [file 41598_2020_78630_MOESM2_ESM.docx]

**DNA metabarcoding of fungal diversity in air and snow of Livingston Island, South Shetland Islands, Antarctica**

Luiz Henrique Rosa, Otávio Henrique Bezerra Pinto, Tina Santl-Temkiv, Peter Convey, Micheline Carvalho-Silva, Carlos Augusto Rosa and Paulo EAS Câmara

**Supplementary Table 2**. Ecological profile at species level of the fungi recovered from air and snow from Livingston Island, South Shetland Islands, Antarctica.

| **Taxa** | **Trophic mode** | **Guild** | **Reference** |
| --- | --- | --- | --- |
| *Pseudogymnoascus roseus* | Saprotrophic | Soil saprotroph | FunGuild^36^ |
| *Lecidea cancriformis* | Symbiotic | Lichenized | Ruprecht *et al.* (2010)^82^ |
| *Austroplaca darbishirei* | Symbiotic | Lichenized | Søchting *et al.* (2014)^83^ |
| *Thelebolus globosus* | Saprotrophic | Dung saprotroph | de Hoog *et al.* (2005)^84^ |
| *Penicillium polonicum* | Saprotrophic | Mycotoxigenic | Khalil *et al.* (2019)^85^ |
| *Pseudogymnoascus appendiculatus* | Saprotrophic | Undefined saprotroph | Rice and Currah (2006)^86^ |
| *Lecanora physciella* | Symbiotic | Lichenized | Ruprecht *et al.* (2012)^87^ |
| *Cleistothelebolus nipigonensis* | Saprotrophic | Dung saprotroph | Malloch and Cain (2011)^88^ |
| *Chalara pseudoaffinis* | Saprotrophic | Wood saprotroph | Koukol (2011)^89^ |
| *Neoascochyta paspali* | Pathogenic-Saprotrophic | Plant pathogen-Wood saprotroph | Johnston (1981)^90^ |
| *Paraconiothyrium africanum* | Pathogenic | Plant pathogen | Damm *et al.* (2008)^91^ |
| *Phaeoacremonium hungaricum* | Pathogenic | Plant pathogen | Essakhi *et al.* (2008)^92^ |
| *Schwanniomyces polymorphus* | No data available | No data available | - |
| *Septoriella hirta* | Saprotrophic | Plant pathogen | Marin-Felix *et al.* (2019)^93^ |
| *Penicillium fluviserpens* | Pathogenic-Saprotrophic | Plant pathogen-Wood saprotroph | Nguyen *et al.* (2020)^94^ |
| *Saccharomyces cerevisiae* | Saprotrophic | Undefined saprotroph | FunGuild^36^ |
| *Aspergillus niger* | Pathogenic-Saprotrophic | Wood saprotroph | FunGuild^36^ |
| *Volucrispora graminea* | Saprotrophic | Plant pathogen-Wood saprotroph | Ingold *et al.* (1968)^95^ |
| *Aspergillus sydowii* | Pathogenic | Animal pathogen | FunGuild^36^ |
| *Penicillium steckii* | Saprotrophic | Soil saprotroph | Samson *et al.* (2004)^96^ |
| *Leptosphaeria sclerotioides* | Pathogenic | Plant pathogen | de Gruyter *et al.* (2012)^97^ |
| *Buellia russa* | Symbiotic | Lichenized | Kim *et al.* (2006)^98^ |
| *Penicillium brasilianum* | Pathogenic | Animal pathogen | FunGuild^36^ |
| *Phaeosphaeria dennisiana* | Pathogenic | Plant pathogen | Rosa *et al.* (2009)^99^ |
| *Pseudallescheria ellipsoidea* | Pathogenic | Human opportunistic | Zouhair *et al.* (2013)^100^ |
| *Lodderomyces elongisporus* | Pathogenic | Animal pathogen | FunGuild^36^ |
| *Candida tropicalis* | Pathogenic-Saprotrophic-Symbiotic | Animal pathogen-Endophyte-Undefined saprotroph | FunGuild^36^ |
| *Penicillium paxilli* | Saprotrophic | Wood saprotroph | Cole *et al.* (1974)^101^ |
| *Paraphoma fimeti* | Saprotrophic | Plant pathogen | FunGuild^36^ |
| *Colletotrichum annellatum* | Pathogenic | Plant pathogen | Damm *et al.* (2012)^102^ |
| *Polysporina subfuscescens* | Symbiotic | Lichenized | Knudsen and Kocourkavá (2008)^103^ |
| *Penicillium astrolabium* | Pathogenic | Plant pathogen | Serra and Peterson (2007)^104^ |
| *Cladosporium halotolerans* | Pathogenic | Animal pathogen | FunGuild^36^ |
| *Lecanora contractula* | Symbiotic | Lichenized | Śliwa *et al.* (2012)^105^ |
| *Penicillium cairnsense* | Saprotrophic | Soil saprotroph | Houbraken and Samson (2011)^106^ |
| *Cladonia rei* | Symbiotic | Lichenized | Syrek and Kukwa (2008)^107^ |
| *Neodevriesia capensis* | Pathogenic | Plant pathogen | Crous *et al.* (2014)^108^ |
| *Penicillium sumatraense* | Pathogenic | Plant pathogen | Taieb *et al.* (2019)^109^ |
| *Mycosphaerella tassiana* | Pathogenic | Plant pathogen | FunGuild^36^ |
| *Fusarium solani* | Pathogenic-Saprotrophic-Symbiotic | Animal pathogen-Endophyte-Plant pathogen-Wood saprotroph | FunGuild^36^ |
| *Placopsis contortuplicata* | Symbiotic | Lichenized | Schmitt *et al.* (2003)^110^ |
| *Bacidina arnoldiana* | Symbiotic | Lichenized | Wirth (1994)^111^ |
| *Penicillium citrinum* | Pathogenic | Plant pathogen | FunGuild^36^ |
| *Zymoseptoria verkleyi* | Pathogenic | Plant pathogen | Crous *et al.* (2012)^112^ |
| *Aspergillus penicillioides* | Pathogenic | Animal pathogen | FunGuild^36^ |
| *Pichia kluyveri* | Pathogenic | Animal pathogen | FunGuild^36^ |
| *Fusarium asiaticum* | Pathogenic | Plant pathogen | Gale *et al.* (2002)^113^ |
| *Malassezia restricta* | Pathogenic | Animal pathogen | FunGuild^36^ |
| *Malassezia globosa* | Pathogenic | Animal pathogen | FunGuild^36^ |
| *Rhodotorula diobovata* | Saprotrophic | Soil saprotroph | Civiero *et al.* (2018)^114^ |
| *Rhodotorula mucilaginosa* | Pathogenic | Animal pathogen | FunGuild^36^ |
| *Leucosporidiella creatinivora* | Saprotrophic | Soil saprotroph | FunGuild^36^ |
| *Heterochaete shearii* | Saprotrophic | Wood saprotroph | Dueñas (2005)^115^ |
| *Calyptella capula* | Saprotrophic | Wood saprotroph | Lee *et al.* (2009)^116^ |
| *Pluteus ephebeus* | Saprotrophic | Wood saprotroph | Gillet (1876)^117^ |
| *Malassezia equina* | Pathogenic | Animal pathogen | FunGuild |
| *Vishniacozyma victoriae* | Pathogenic-Saprotrophic | Animal pathogen-Litter saprotroph | FunGuild^36^ |
| *Phanerochaete sordida* | Saprotrophic | Wood saprotroph | Volobuev *et al.* (2015)^118^ |
| *Hyphodontia microspora* | Saprotrophic | Wood saprotroph | Yurchenko and Wu (2016)^119^ |
| *Peniophora laxitexta* | Saprotrophic | Wood saprotroph | Majul *et al.* (2020)^120^ |
| *Vishniacozyma tephrensis* | Saprotrophic | Soil saprotroph | Li *et al.* (2020)^121^ |
| *Vanrija humicola* | Saprotrophic | Soil and mushroom saprotroph | Kurtzman *et al.* (2011)^122^ |
| *Malassezia sympodialis* | Saprotrophic | Animal pathogen | FunGuild^36^ |
| *Mortierella fimbricystis* | Saprotrophic-Symbiotic | Endophyte-Litter Saprotroph-Soil Saprotroph-Undefined Saprotroph | Cannon and Kirk (2007)^123^, Tedersoo *et al.* (2014)^124^, Purahong *et al.* (2016)^125^ |
| *Mortierella gamsii* | Saprotrophic-Symbiotic | Endophyte-Litter Saprotroph-Soil Saprotroph-Undefined Saprotroph | Cannon and Kirk (2007)^123^, Tedersoo *et al.* (2014)^124^, Purahong *et al.* (2016)^125^ |
| *Mortierella parvispora* | Saprotrophic-Symbiotic | Endophyte-Litter Saprotroph-Soil Saprotroph-Undefined Saprotroph | Cannon and Kirk (2007)^123^, Tedersoo *et al.* (2014)^124^, Purahong *et al.* (2016)^125^ |
| *Mortierella alpina* | Saprotrophic-Symbiotic | Endophyte-Litter Saprotroph-Soil Saprotroph-Undefined Saprotroph | Cannon and Kirk (2007)^123^, Tedersoo *et al.* (2014)^124^, Purahong *et al.* (2016)^125^ |
| *Mortierella elongatula* | Saprotrophic-Symbiotic | Endophyte-Litter Saprotroph-Soil Saprotroph-Undefined Saprotroph | Cannon and Kirk (2007)^123^, Tedersoo *et al.* (2014)^124^, Purahong *et al.* (2016)^125^ |
| *Mortierella turficola* | Saprotrophic-Symbiotic | Endophyte-Litter Saprotroph-Soil Saprotroph-Undefined Saprotroph | Cannon and Kirk (2007)^123^, Tedersoo *et al.* (2014)^124^, Purahong *et al.* (2016)^125^ |
