# Supplementary material for: DNA metabarcoding of fungal diversity in air and snow of Livingston Island, South Shetland Islands, Antarctica
Source: Sci Rep. 2020 Dec 11;10:21793. doi: 10.1038/s41598-020-78630-6 (PMC7733504; doi:10.1038/s41598-020-78630-6)
Supplement: Supplementary file 3 — Supplementary Information. [file 41598_2020_78630_MOESM3_ESM.docx]

**DNA metabarcoding of fungal diversity in air and snow of Livingston Island, South Shetland Islands, Antarctica**

Luiz Henrique Rosa, Otávio Henrique Bezerra Pinto, Tina Santl-Temkiv, Peter Convey, Micheline Carvalho-Silva, Carlos Augusto Rosa and Paulo EAS Câmara

**Supplementary Table 3**. Ecological profile at generic level of the fungi recovered from air and snow from Livingston Island, South Shetland Islands, Antarctica.

| **Taxa** | **Trophic mode** | **Guild** | **Reference** |
| --- | --- | --- | --- |
| *Cladosporium* | Pathogenic-Saprotrophic-Symbiotic | Animal pathogen-Endophyte-Lichen parasite-Plant pathogen-Wood saprotroph | FunGuild^36^ |
| *Pseudogymnoascus* | Pathogenic-Saprotrophic-Symbiotic | Animal pathogen-Soil saprotroph | FunGuild^36^ |
| *Meyerozyma* | Symbiotic-Pathogenic | Epiphyte-Animal pathogen | FunGuild^36^ |
| *Penicillium* | Saprotrophic | Dung saprotroph-Undefined saprotroph-Wood saprotroph | FunGuild^36^ |
| *Hanseniaspora* | Pathogenic | Animal pathogen | FunGuild^36^ |
| *Septoriella* | Pathogenic | Plant pathogen | Marin-Felix *et al.* (2019)^93^ |
| *Cyberlindnera* | Pathogenic | Animal pathogen | FunGuild^36^ |
| *Mitrulinia* | Saprotrophic | Undefined saprotroph | FunGuild^36^ |
| *Pestalotiopsis* | Pathogenic | Plant pathogen | FunGuild^36^ |
| *Debaryomyces* | Saprotrophic | Undefined saprotroph | FunGuild^36^ |
| *Lecidea* | Symbiotic | Lichenized | FunGuild^36^ |
| *Colletotrichum* | Pathogenic-Symbiotic | Endophyte-Plant pathogen | FunGuild^36^ |
| *Rhizoscyphus* | Pathogenic-Saprotrophic-Symbiotic | Bryophyte parasite-Ectomycorrhizal-Ericoid mycorrhizal-Undefined saprotroph | FunGuild^36^ |
| *Aspergillus* | Pathogenic-Saprotrophic-Symbiotic | Animal Pathogen-Endophyte-Plant Saprotroph-Soil Saprotroph-Undefined Saprotroph-Wood Saprotroph | FunGuild^36^ |
| *Pseudallescheria* | Saprotrophic | Undefined saprotroph | FunGuild^36^ |
| *Yamadazyma* | Pathogenic | Animal pathogen | FunGuild^36^ |
| *Trichoderma* | Pathogenic-Saprotrophic-Symbiotic | Endophyte-Epiphyt-Fungal parasite-Plant pathogen-Wood saprotroph | FunGuild^36^ |
| *Pseudeurotium* | Saprotrophic | Undefined Saprotroph | FunGuild^36^ |
| *Cystodendron* | Pathogenic | Null | FunGuild^36^ |
| *Neopestalotiopsis* | Pathogenic | Plant pathogen | Solarte *et al.* (2018)^126^ |
| *Schwanniomyces* | Pathogenic | Animal pathogen | FunGuild^36^ |
| *Sarocladium* | Saprotrophic | Undefined saprotroph | FunGuild^36^ |
| *Usnea* | Saprotrophic | Lichenized | FunGuild^36^ |
| *Malassezia* | Pathogenic-Saprotrophic | Animal pathogen-Undefined saprotroph | FunGuild^36^ |
| *Marasmius* | Pathogenic-Saprotrophic-Symbiotic | Endophyte-Plant pathogen-Undefined saprotroph | FunGuild^36^ |
| *Gymnopus* | Saprotrophic | Undefined saprotroph | FunGuild^36^ |
| *Mortierella* | Saprotrophic-Saprotrophic | Endophyte-Litter saprotroph-Soil saprotroph-Undefined saprotroph | FunGuild^36^ |
| *Densospora* | Saprotrophic | Ectomycorrhizal | FunGuild^36^ |
