# Supplementary material for: DNA metabarcoding of fungal diversity in air and snow of Livingston Island, South Shetland Islands, Antarctica
Source: Sci Rep. 2020 Dec 11;10:21793. doi: 10.1038/s41598-020-78630-6 (PMC7733504; doi:10.1038/s41598-020-78630-6)
Supplement: Supplementary file 4 — Supplementary References. [file 41598_2020_78630_MOESM4_ESM.docx]

**References**

82. Ruprecht, U. et al. Diversity of *Lecidea* (*Lecideaceae*, *Ascomycota*) species revealed by molecular data and morphological characters. *Antarct. Sci.* **22**, 727-741 (2010).

83. Søchting, U. et al. *Charcotiana* and *Amundsenia*, two new genera in *Teloschistaceae* (lichenized *Ascomycota*, subfamily *Xanthorioideae*) hosting two new species from continental Antarctica, and *Austroplaca frigida*, a new name for a continental Antarctic species. *The Lichenologist* **46**, 763-782 (2014).

84. [Khalil](https://www.sciencedirect.com/science/article/pii/S1878818119308953#!), A.M.A. Occurrence of toxigenic *Penicillium polonicum* in retail green table olives from the Saudi Arabia market. *Biocatal. Agric. Biotechnol.* [21](https://www.sciencedirect.com/science/journal/18788181/21/supp/C), 101314 (2019).

85. Rice, A.V., Currah, R.S. Two new species of *Pseudogymnoascus* with *Geomyces* anamorphs and their phylogenetic relationship with *Gymnostellatospora*. *Mycologia* **98**, 307-318 (2006).

86. Ruprecht, U. et al. Insights into the Diversity of *Lecanoraceae* (*Lecanorales*, *Ascomycota*) in continental Antarctica (Ross Sea region). *Nova Hedwigia* **94**, 287-306 (2012).

87. [Malloch](https://www.researchgate.net/scientific-contributions/9926755-David-Malloch?_sg%5B0%5D=Tbk56nsHvMXCSQPizHMBafaTB6kvbb8DB4iBKNKYFRJ3KCiYfEb6mwxtL1MFJnh_zbnGfV0.9hvPRVnzfXfMsWzc0tEmOkS-2aRxFUzEGcn9eTMzojrf--eTiSrIK4F4QjBpFhu-MG-V1g0FQtzdvHH1RPr6qw&_sg%5B1%5D=47AeUktkI1DNpBNENH8d9zeZekECADciioPaS-NPR9LUlji9EWXAxVxxPDBB4XKHihu5A4g.Hwpab9PK8NkwhEYDkxzYhHC6Xd1uU8M4hVmGsYxSmaINN0ecJUeaOivZzBA1E8XB366NUVoYbrPT7Eu6Y9GGyA), D., [Cain](https://www.researchgate.net/scientific-contributions/R-F-Cain-2009241758?_sg%5B0%5D=Tbk56nsHvMXCSQPizHMBafaTB6kvbb8DB4iBKNKYFRJ3KCiYfEb6mwxtL1MFJnh_zbnGfV0.9hvPRVnzfXfMsWzc0tEmOkS-2aRxFUzEGcn9eTMzojrf--eTiSrIK4F4QjBpFhu-MG-V1g0FQtzdvHH1RPr6qw&_sg%5B1%5D=47AeUktkI1DNpBNENH8d9zeZekECADciioPaS-NPR9LUlji9EWXAxVxxPDBB4XKHihu5A4g.Hwpab9PK8NkwhEYDkxzYhHC6Xd1uU8M4hVmGsYxSmaINN0ecJUeaOivZzBA1E8XB366NUVoYbrPT7Eu6Y9GGyA), R.F. Four new genera of cleistothecial *Ascomycetes* with hyaline ascospores. *Can. J. Bot.* **49**, 847-854 (2011).

88. Koukol, O. New species of *Chalara* occupying coniferous needles. *Fungal Div*. **49**, 75-91 (2011).

89. [Johnston, P.R. *Phoma* on New Zealand grasses and pasture legumes. *N. Z. J. Bot.* 19, 173-186](javascript:void(0)) (1981).

90. Damm, U. et al. novel *Paraconiothyrium* species on stone fruit trees and other woody hosts. *Persoonia* **20**, 9-17 (2008).

91. [Essakhi](https://www.researchgate.net/scientific-contributions/35907969-S-Essakhi?_sg%5B0%5D=fyRCkwA6J-x1TVLjJAjwkZXRpwShCrUZj9zal0FEc8DS5lyERc24ngOg2D6848aYDEppIv4.bCtlglOcDodjLGZgaDwrTyih9smumn68EVOreBqd85KP2GS1oiLLw_syjwFPMui7-dKBJ0dtxrMTJh8wsyXutA&_sg%5B1%5D=9KQe-Z-BV5EPnd8FqOhJQclYESy8RKl5_yu6evPRre-EDaZ9Pz88Fs0yUJNLHf-aM7Pwk_w.2Y3rQ6PG3ReFYUv1z_NhWMi17z7xy7-wdOZ0pzGC9DdI6TQNCKVfZPBXiVsKKUF4JL4N493USp55GtGAmrPw9g), S. et al. Molecular and phenotypic characterization of novel *Phaeoacremonium* species associated with Petri disease and esca of grapevine. *Persoonia* **21**, 119-34 (2008).

92. [Marin-Felix](https://www.sciencedirect.com/science/article/pii/S0166061619300089?via%3Dihub#!), Y. Genera of phytopathogenic fungi: GOPHY 3. [*Stud. Mycol*.](https://www.sciencedirect.com/science/journal/01660616) [94](https://www.sciencedirect.com/science/journal/01660616/94/supp/C), 1-124 (2019).

93. Nguyen, T.T.T. et al. Five New Records of the Family *Aspergillaceae* in Korea, *Aspergillus europaeus*, *A. pragensis*, *A. tennesseensis*, *Penicillium fluviserpens*, and *P. scabrosum*. *Mycobiology* **48**, 81-94 (2020).

94. Ingold, C.T. et al. *Volucrispora graminea* sp. nov. *TBMS* **51**, 325-329 (1968).

95. Samson, R.A., Hoekstra, E.S., Frisvad, J.C. Introduction to food- and airborne fungi, 7th edn. Centraalbureau voor Schimmelcultures, Utrecht (2004).

96. de Gruyter, J. et al. Redisposition of phoma-like anamorphs in *Pleosporales*. *Stud. Mycol.* **75**, 1-36 (2012).

97. Kim, J.H. et al. Lichen flora around the Korean AntarcticScientific Station, King George Island, Antarctic. *J. Microbiol*. **44**, 480-91 (2006).

98. Rosa, L.H. et al. Endophytic fungi associated with the Antarctic Grass *Deschampsia antarctica* Desv. (Poaceae). *Polar Biol.* **32**, 161-167 (2009).

99. Zouhair, R. et al. Distribution of the different species of the *Pseudallescheria boydii*/*Scedosporium apiospermum* complex in French patients with cystic fibrosis. *Med. Mycol.* **51**, 603-613 (2013).

100. Cole, R.J. et al. A new tremorgenic metabolite from Penicillium paxilli. Can. J. Microbiol. **20**, 1159–1162 (1974).

101. [Damm](https://www.sciencedirect.com/science/article/pii/S0166061614600750#!), U. The Colletotrichum boninense species complex. [*Stud. Mycol.*](https://www.sciencedirect.com/science/journal/01660616) [**73**](https://www.sciencedirect.com/science/journal/01660616/73/supp/C), 1-36 (2013).

102. Knudsen, K., Kocourková, J. A study of lichenicolous species of *Polysporina* (*Acarosporaceae*). *Mycotaxon* **105**, 149-164 (2008).

103. Serra, R., Peterson, S.W. *Penicillium astrolabium* and *Penicillium neocrassum*, two new species isolated from grapes and their phylogenetic placement in the *P. olsonii* and *P. brevicompactum* clade. *Mycologia* **99**, 78-87 (2007).

104. Śliwa, L. et al. Are widespread morphospecies from the *Lecanora dispersa* group (lichen-forming *Ascomycota*) monophyletic? *The Bryologist* **115**, 265-277 (2012).

105. Houbraken, J., Samson, R.A. Phylogeny of *Penicillium* and the segregation of *Trichocomaceae* into three families. *Stud. Mycol.* **70**, 1-51 (2011).

106. Syrek, M., Kukwa, M. Taxonomy of the lichen *Cladonia rei* and its status in Poland. *Biologia* **63**, 493-497 (2008).

107. Crous, P.W. et al. Fungal Planet description sheets: 214–280. *Persoonia* **32**, 184-306 (2014).

108. Taieb, K.H. Phytopathogenic and antagonistic potentialities of fungiassociated with pistachio bark beetle, *Chaetoptelius vestitus* (Coleoptera, Curculionidae), infesting pistachio (*Pistacia vera*) in Tunisia. *J. Appl. Microbiol.* **126**, 1821-1834 (2019).

109. Schmitt, I. et al. Phylogeny of the lichen genus *Placopsis* and its allies based on Bayesian analyses of nuclear and mitochondrial sequences. *Mycologia* **95**, 827-35 (2003)

110. [Wirth, V. Checkliste der Flechten und flechtenbewohnenden Pilze Deutschlands - eine Arbeitshilfe. Stuttgarter Beiträge zur Naturkunde 517, 1-63](javascript:void(0)) (1994).

111. Crous, et al. Fungal Planet description sheets: 128–153. [*Persoonia*](https://www.ingentaconnect.com/content/nhn/pimj) **29**, 146-201 (2012).

112. Gale, L.R. et al. Kistler Population analysis of *Fusarium graminearum* from wheat fields in eastern China. *Phytopathology* **92**, 1315-1322 (2002).

113. [Civiero](https://www.ncbi.nlm.nih.gov/pubmed/?term=Civiero%20E%5BAuthor%5D&cauthor=true&cauthor_uid=29966334), E. et al. Physiological and phylogenetic characterization of Rhodotorula diobovata DSBCA06, a nitrophilous yeast. *Biology* **7**, 39 (2018).

114. [Dueñas](https://www.researchgate.net/profile/Margarita_Duenas?_sg%5B0%5D=_B2GdMOcV-5PkWwYleOIGRabonM1L9rgd4Zcqf89zftoRLg47b6WzUvcS3gky-t6zarT2V4.2j6Z_hdH_LtrEv0q7dOwcgPaK6nDzzI45bo_1tL-aHtMDEjyidHvU4qOVSxcJZK6JHtkpQYC5ESwTdANxwkK1w&_sg%5B1%5D=aOYl8L5kz46Irr2Bqd01Ya4VbQ-5rBwbUE3nK_YI56WH3x7U6LvjhRtQcAB71q9GTtzJHss.ShIRwEHgm-etDPWLUe4nR7KVKPVSYlQ5dDV5C7bFdAbSeOlWlFlWY-z_TEOlv5_e4QSXWq8OScTPbBUKSh0erg), M. New and interesting Iberian heterobasidiomycetous fungi. I. [*Nova Hedwigia*](https://www.ingentaconnect.com/content/schweiz/novh) **81**, 177-198 (2005).

115. Lee, et al. New record of the genus *Calyptella* from Korea. *Mycobiology* **37**, 1-4 (2009).

116. [Gillet, C.C. Les Hyménomycètes ou description de tous les champignons qui croissent en France. 177-560](javascript:void(0)) (1876).

117. Volobuev, S. et al. The *Phanerochaete sordida* group (*Polyporales*, *Basidiomycota*) in temperate Eurasia, with a note on *Phanerochaete pallida*. *Mycol. Prog.* **14**, 80 (2015).

118. Yurchenko, E., Wu, S.H. A key to the species of *Hyphodontia* sensu lato. *MycoKeys* **12**, 1-27 (2016).

119. Majul, L. et al. High dye removal capacity of *Peniophora laxitexta* immobilized in a combined support based on polyurethane foam and lignocellulosic substrates. *Environ. Technol.* https://doi.org/10.1080/09593330.2020.1801851 (2020).

120. Li, A.H. et al. Diversity and phylogeny of basidiomycetous yeasts from plant leaves and soil: Proposal of two new orders, three new families, eight new genera and one hundred and seven new species. [*Stud. Mycol.*](https://www.sciencedirect.com/science/journal/01660616) [**96**](https://www.sciencedirect.com/science/journal/01660616/96/supp/C), 17-140(2020).

121. Kurtzman, C.P., Fell, J,W., Boekhout, T. The yeasts: a taxonomic study. Elsevier, Amsterdam (2011).

122. Cannon, P.F., Kirk, P.M. Fungal Families of the World. CAB International, Cambridge (2007).

123. Tedersoo, L. et al. Global diversity and geography of soil fungi. *Science* **346**, 1256688 (2014).

# 124. Purahong, W. et al. Life in leaf litter: novel insights into community dynamics of bacteria and fungi during litter decomposition. *Mol. Ecol.* 25, 4059-407 (2016).

# 125. [Solarte](https://apsjournals.apsnet.org/doi/full/10.1094/PDIS-01-17-0068-RE), F. et al. Diversity of *Neopestalotiopsis* and *Pestalotiopsis* spp., causal agents of guava scab in Colombia. *Plant Dis*. 104, 49-59 (2018).
